# Influence of the Fabrication Accuracy of Hot-Embossed PCL Scaffolds on Cell Growths

**DOI:** 10.3389/fbioe.2020.00084

**Published:** 2020-02-14

**Authors:** Tania Limongi, Elisabetta Dattola, Cirino Botta, Maria Laura Coluccio, Patrizio Candeloro, Maria Cucè, Bernadette Scopacasa, Maria Eugenia Gallo Cantafio, Costantino Davide Critello, Salvatore Andrea Pullano, Antonino S. Fiorillo, Pierosandro Tagliaferri, Pierfrancesco Tassone, Ernesto Lamanna, Enzo Di Fabrizio, Gerardo Perozziello

**Affiliations:** ^1^Department of Applied Science and Technology, Polytechnic University of Turin, Turin, Italy; ^2^Department of Experimental and Clinical Biomedical Sciences “Mario Serio”, University of Florence, Florence, Italy; ^3^Department of Experimental and Clinical Medicine, University of Magna Graecia, Catanzaro, Italy; ^4^Department of Health Sciences, University of Magna Graecia, Catanzaro, Italy; ^5^King Abdullah University of Science and Technology (KAUST), Thuwal, Saudi Arabia

**Keywords:** polycaprolactone, demolding force, microstructured scaffolds, hot embossing, cell viability

## Abstract

Polycaprolactone (PCL) is a biocompatible and biodegradable polymer widely used for the realization of 3D scaffold for tissue engineering applications. The hot embossing technique (HE) allows the obtainment of PCL scaffolds with a regular array of micro pillars on their surface. The main drawback affecting this kind of micro fabrication process is that such structural superficial details can be damaged when detaching the replica from the mold. Therefore, the present study has focused on the optimization of the HE processes through the development of an analytical model for the prediction of the demolding force as a function of temperature. This model allowed calculating the minimum demolding force to obtain regular micropillars without defects. We demonstrated that the results obtained by the analytical model agree with the experimental data. To address the importance of controlling accurately the fabricated microstructures, we seeded on the PCL scaffolds human stromal cell line (HS-5) and monocytic leukemia cell line (THP-1) to evaluate how the presence of regular or deformed pillars affect cells viability. *In vitro* viability results, scanning electron and fluorescence microscope imaging analysis show that the HS-5 preferentially grows on regular microstructured surfaces, while the THP-1 on irregular microstructured ones.

## Introduction

The recent progress in the field of micro- and nanotechnologies (Perozziello et al., 2014, 2015) along with latest cells culture techniques (Perozziello et al., 2012) have allowed the development of engineered materials and devices that can be used to monitor aspects of human health improving medical diagnosis and therapy. These novel materials characterized by micro- and nano- structured textures represent new solutions for satisfying the wide range of needs arising from the diagnosis and treatment of several human pathologies. This technological approach has opened the door also to successfully 3D cells culture, tissue engineering and regenerative medicine applications. Research in the field of biomaterials has made the development of ideal substitutes for repair and restore the physiological function of tissues such as muscle, bone, cartilage, skin brain, and nerves (Park et al., 2007; Dvir et al., 2011; Zhu et al., 2014; Limongi et al., 2017a). One of the most investigated approaches is to implement biomaterials which act as temporary sustain (scaffolds) in which to seed the cells for tissue regrowth. The main purpose of using scaffolds is to assist the replication of the original tissue environment, which is mainly constituted by extracellular matrix (ECM), an intricate network of macromolecules such as collagens, elastin, and glycosaminoglycans (Fomovsky et al., 2010). In this context, the biomimetic scaffold (O’brien, 2011; Khademhosseini and Langer, 2016) applications range from tissue engineering, prosthesis, analytical devices, microfluidic devices, to the *in vitro* diagnostics and screening applicattions. In tissue engineering field, the scaffolds represent a three-dimensional (3D) structure able to assist and tune cell/tissue morphogenesis (Zhang and Webster, 2009). Therefore, a wide range of researches aim at the design of the ideal device considering the obtainment of the best mechanoelastic properties, topography, and biocompatibility. Recent studies have shown how a proper support influences cell adhesion and proliferation (Takeda et al., 2016) by promoting the construction of physiological 3D networks. The presence of specific micro- nano structures molded on the surface of such devices makes cell adhesion evident to the side walls of fabricated micro-pillars and consequently allows the formation of a suspended 3D cell culture (Gentile et al., 2010; Chung et al., 2013; Limongi et al., 2013). Considering its biocompatibility and mechanical properties, Polycaprolactone (PCL) has the requirements suitable for the production of scaffolds for long-term application since its biodegradation is lower than other polymers (Leja and Lewandowicz, 2010). Moreover, its wettability is such as to ensure a proper balance between cell-cell and cell-substrate interactions allowing the formation of healthy cell networks on the scaffold. PCL has been approved by the Food and Drug Administration (FDA) in specific biomedical use (Woodruff and Hutmacher, 2010). Its slow degradation time makes it suitable for clinical application as in the case of Osteoplug^TM^, a bioresorbable implant used to neurosurgery burr-hole trephination covering and in the case of Neurolac® able to assist peripheral nerve reconstruction. In Causa et al. (2006) authors described how a PCL scaffold, reinforced with hydroxyapatite (HA) particles, is a good solution for the regeneration of bones promoting the proliferation of human osteoblasts and for the regeneration of cartilage tissue (Wei et al., 2015; Zhang et al., 2016). Regarding new effective 3D cell culture applications, Palomeras et al. (2016) used the PCL scaffolds for the *in vitro* growth of cancer stem cells (CSCs). These cell type represents a minor subpopulation in solid tumors able to drive tumor progression and metastatization. CSCs are difficult to culture in 2D systems and Palomeras et al. (2016) successfully demonstrated their enrichment due to seeding them on a PCL scaffold. Their 3D PCL scaffolds resulted a useful way to enrich and propagate CSCs for further investigation targeted to this malignant subpopulation. Last but not least, several PCL nanofibres have been successfully loaded with 20(S)-protopanaxadiol, doxorubicin and other active molecules for both *in vitro* and *in vivo* anti-tumor activity applications (Cabeza et al., 2017; Liu et al., 2018).

It was shown that Hot Embossing (HE) (Heckele et al., 1998; Becker and Heim, 2000; Peng et al., 2013) is an effective replication technology to build polymer devices at micro and nano-scale. HE allows also the realization of specific patterns on the surface of different biomaterials for tissue engineering and regenerative medicine applications (Charest et al., 2004; Truskett and Watts, 2006; Moroni and Lee, 2009; Choi et al., 2012; Brown et al., 2013; Lima et al., 2014; Yan et al., 2017). This technique can be applied to PCL (Limongi et al., 2013, 2015), which is a thermoplastic material. Although this technology allows the fabrication of micro-devices with high precision, it carries on, unfortunately, some disadvantages linked to structural defects that may occur when disconnecting the replica from the mold. Thermal stress, strong adhesion or friction can negatively affect biomimetic surface fabrication and the resulting structural defects can impair cell adhesion and proliferation.

The purpose of this work is to understand which parameters affect the micro texture of HE structures, and how imperfections on microtextured scaffolds could affect cells’ viability. The HE process was examined through the implementation of an analytical model able to predict the demolding force. By using this kind of fabrication technique, the replica microstructures stuck on the silicon mold and a demolding force should be applied to detach the replica from the mold. If this force produces stretching pressures on the just realized polymer microstructures, these will be deformed. In this work, we investigated the parameters related to the demolding force and the possibility to minimize them. In particular, the developed model was able to predict the demolding force as a function of the temperature of the mold and the replica. It was validated by using an experimental set-up to allow the measure of the demolding force of the embossed PCL microstructures. Finally, preliminary *in vitro* experiments were performed by growing monocytes (THP-1) and mesenchymal stem cells (HS-5) on both deformed and regular PCL microstructures to demonstrate how the micro texture of scaffolds could be crucial for regenerative medicine and tissue-engineering applications.

## Theoretical Model

**FIGURE 1:**
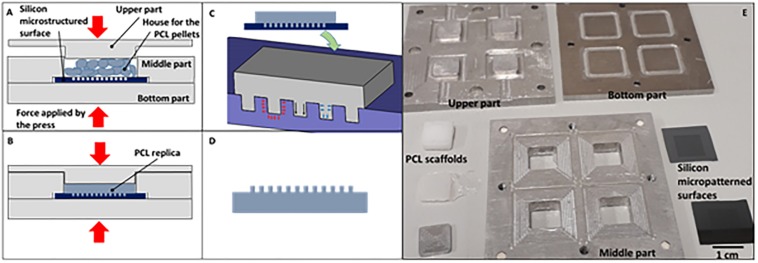
Scheme of the aluminum mold and the hot embossing (HE) process: **(A)** heating up of the aluminum mold and the PCL pellets; **(B)** hot embossing of the PCL replica; **(C)** cooling down of the PCL replica and the silicon microstructured substrate [the tensions acting between the replica and the mold due to adhesion (red arrows), friction (black arrows), thermal expansion (blue arrows) are also reported]; **(D)** PCL replica removed from the mold; **(E)** Picture of the different part composing the aluminum mod and PCL scaffolds.

In order to optimize the HE process (Figures 1A–D), we developed an analytical model based on a precedent proposed by Omar et al. (2014). The presented analytical model allows predicting the demolding force after a HE process. This force should be carefully taken in account to minimize the deformation of the replicated structures. In fact, the demolding force should be minimized in order to decrease the stretching of the microstructures reproduced on the replicas stuck in the mold. In particular, the study adopted to develop the proposed model, allowed the prediction of the demolding force as a function of the mold and replica temperature. The model was based on the assumption that the mold was made of an array of circular microcavities, and a replica, reproducing an array of micropillars, made of PCL. It was assumed that the demolding force (F*_*Dem*_*) was composed of three components: adhesion (F*_*Ad*_*), deformation (F*_*Def*_*), and friction forces (F*_*Attr*_*) (Figure 1C).

(1) $$F_{D\,e\,m}=F_{A\,d}+F_{D\,e\,f}+F_{A\,t\,t\,r}$$

It is known that the pressure generated during the embossing creates adhesion between the horizontal surfaces at the base of the mold microcavities and the tips of the micropillars of the replica.

The resulting adhesion force was calculated as a function of the adhesion surface (A) and adhesion strength (*s*_*ad*_):

(2) $$F_{A\,d}=\sigma_{a\,d}\times A$$

**FIGURE 2:**
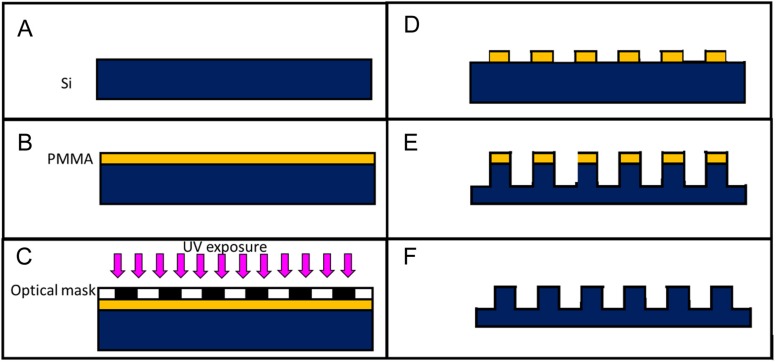
Sketch of the mold fabrication process: a silicon substrate (Si) is cleaned in a ultrasonic bath **(A)** a photoresist (PMMA) is spun on top of the silicon substrate **(B)** the sample is exposed to UV after being masked **(C)** the exposed photoresist is dissolved in a solvent **(D)** the micropillars are dry-etched in the silicon sample **(E)** the remaining photoresist is removed with acetone **(F)**.

In turn, the adhesion strength was related to the material properties and interfaces and it was calculated by considering the change of the adhesion force of a material subjected to temperature variations (Kendall, 1973). The link between adhesion strength and temperature variations is due to the change in the volume of the mold and the substrate. A temperature decrease, from the values of the embossing temperature to those of the demolding temperature causes a mold and replica shrinkage. If the mold and the replica undergo shrinkage, there is a reduction of the interface area and therefore the adhesion force decreases. The stress due to the shrinkage involves labor and causes the deformation of the system. The energy dissipated due to the shrinkage reduces the initial adhesion energy at zero shrinkage (γ, due to molecular attraction). The adhesion tension can therefore be calculated as,

(3) $$\sigma_{a\,d}={\left[\frac{2\,K}{t_{p}}\cdot \left(\gamma -\frac{t_{p}\cdot K\cdot \varepsilon^{2}}{2}\right)\right]}^{1/2}$$

where *t*_*p*_ is the height of the micropillars, K is the bulk modulus of the replica,

(4) $$K=\frac{E}{3\,\left(1-2\cdot \nu \right)}$$

and the ε is the thermal shrinkage deformation, calculated with the following equation,

(5) $$\varepsilon =\alpha_{p}\times \Delta \,T$$

where E is the polymer Young modulus, ν is the Poisson ratio, α*_*p*_* is the coefficient of linear thermal expansion of the polymer and ΔT is the thermal variation that involves the shrinkage. The deformation and friction components take in account the deformation that the replica must undergo to be able to slip out of the mold. In fact, the profile of the cavity, reproducing the negative of the micropillars, in the mold, is never completely smooth and uniform; it was due to the particular process by which a certain roughness was manufactured. The roughness causes joints and friction between mold and replica. To determine the force necessary to remove the substrate from the mold it was considered the theory proposed by Colton et al. (2001), modified considering the fact that the molds used in this work were made in Silicon by optical lithography and etching processes (Figure 2).

(6) $$F_{D\,e\,f}=\frac{\delta^{2}}{l\times r_{c}}\cdot \frac{E}{1-\nu }\cdot A_{w}$$

The first part of the equation (6) considers the uneven profile of the cavities. The etching process yields a sinusoidal profile with a peak-to-valley distance (δ) and a peak-to-peak distance (*l*) known; *r*_*c*_ is the hydraulic radius of the cavity, calculated as half of the diameter (*d*) of the circular micropillars. The second part of the equation concerns the deformation of the polymer and the third is the contact area between polymer and the side walls of the cavity (*A*_*w*_). The frictional force is the product of the coefficient of friction (μ), the contact stress on the side walls of the cavities (σ*_*d*_*) and the area of the walls themselves (*A*_*w*_):

(7) $$F_{A\,t\,t\,r}=\mu \cdot \sigma_{d}\cdot A_{w}$$

The contact stress depends on the temperature used during the hot-embossing process. This contribution is given by:

(8) $$\sigma_{d}=\sigma_{a\,d}+\sigma_{b\,d}$$

σ*_*bd*_* is the residual stress due to the fact that the mold and the replica are cooled down before the embossing pressure (p) is released, p is considered constant in each point and is equal to the force applied by the thermal press divided by the surface *A*. In this condition, the friction, the adhesion and pressure counteract the shrinkage. Then, σ*_*bd*_* can be calculated as

(9) $$\sigma_{b\,d}=\sigma_{T}-\left[p+\mu \cdot \left(p+\sigma_{a\,d}\right)\right]$$

The thermal stress (σ*_*T*_*) is a result of the polymer shrinkage during cooling:

(10) $$\sigma_{T}=\left(\alpha_{p}-\alpha_{m}\right)\cdot \left(T_{g}-T_{d}\right)\cdot \frac{E}{1-\nu }$$

where α*_*p*_* is the polymer thermal expansion coefficient and α*_*m*_* is that of the mold, T*_*g*_* is the polymer glass transition temperature, T*_*d*_* is the temperature during demolding. Putting these terms together we were able to calculate the demolding force as a function of the material properties, embossing pressure and, in particular, of the temperature of the mold and replica during the demolding.

## Materials and Methods

### Hot Embossing of the Polycaprolactone Replicas

The HE process for the fabrication of the microstructured PCL (Sigma-Aldrich, Mn 80,000) replicas was performed by using a Lab press (Presstronic P/O/Weber). The molds used for the replica were composed of two parts (Figure 1E). The first is -a silicon microstructured substrate (0.5 mm thick and 1 cm long and wide). The microstructures consisted in a hexagonal array of micro cavities having a diameter of 10 μm, a depth of 10 μm, and a pitch of 20 μm (distance between the microcavities). The second is an aluminum holder reproducing four pockets hosting four silicon microstructured substrates. The silicon substrates were fabricated by optical lithography and deep reactive-ion etching (DRIE) techniques as already described in Limongi et al. (2017b) and schematically represented in Figure 2. These allowed to reproduce micropillars in the replica and the aluminum holder was fabricated by milling. It allowed to host, at the bottom surface, four silicon substrates and to give the same external layout to the polymer replicas (a 4 mm thick dice, 1 cm long and wide). The external layout of the replica allowed fixing it to a set-up by which the demolding force needed to detach the polymer replica from the silicon mold could be measured. To recreate the replicas, PCL pellets were inserted in the mold. Subsequently, the mold was placed inside the press and heated (133°C) to melt the PCL pellets. Then, the temperature was decreased to a value of 68°C, to which the embossing was performed by applying a force of about 4 kN. Mold and replica remained in contact for 30 min until the temperature dropped to a value of 25–29°C. Finally, the mold was opened for extracting the four replicas.

### Measure of the Demolding Force Between the Molds and the Replicas

**FIGURE 3:**
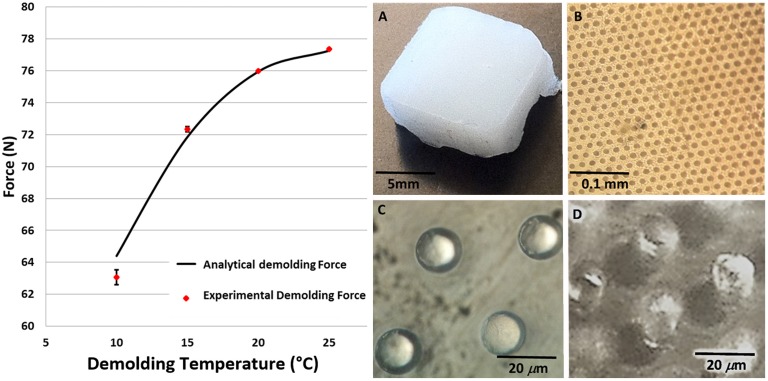
Left panel: calculated and experimental data of the demolding force as a function of the demolding temperature (*p* < 0.05). In the right panel macroscopic view of the PCL scaffolds captured by an optical microscope: **(A)**, low **(B)** and high magnification top view **(C)** and tilted view **(D)**.

After the HE process, the mold was detached from the replica by using an experimental set-up by which we measured the demolding force and the temperature of the replica during the process (Supplementary Material). In addition, during the detachment, the temperature of the replicas was measured by means of a laser thermometer connected to the set-up. Tests, in which the molds were detached from the replicas, were performed in triplicate at different demolding temperatures (25, 20, 15, and 10°C). To cool the replicas to the desired demolding temperature, the demolding tweezer, containing the silicon mold and the PCL replica, were entirely immersed in ice. The temperature was monitored with the laser thermometer (by pointing the laser on the PCL replica) and at the achievement of the desired temperature, the tweezer was removed from ice and inserted into a press connected to the force sensor to run the demolding process. During the demolding, the force and the temperature were monitored. The demolding force corresponded to the value of the force recorded at the instant in which the replica detached from the silicon substrate. This instant could be easily recognized by a rapidly decrease of the force values after the detachment. The results obtained were compared to those calculated by using the developed analytical model. The different parameters used for the equations of the analytical model were reported in the Table 1. The experimental data and the analytical ones were compared graphically (Figure 3, left).

**TABLE 1:** Values of the different parameters used for the analytical model (the Young modulus and Bulk modulus range is due to the fact that different types of PCL were found in literature).

Properties	Symbol	Value		Measure
		**Min**	**Max**	
Height of the microstructures	t_P_	10		μ*m*
Poisson ratio	*v*	0.3		–
Young modulus	E	0.2	0.4	Gpa
Bulk modulus	K	0.2	0.3	Gpa
Thermal expansion coefficient (PCL)	*α_*p*_*	16		10^–5^/°C
Adhesion energy	*γ*	16		mJ/m^2^
Total horizontal contact Area	A	1		cm^2^
Lateral contact area	*Aw*,	3		10^–10^m^2^
Coefficient of friction	μ	0.3		–
Thermal expansion coefficient (Si)	α_*m*_	0.3		10^–5^/°C
Glass transition temperature	Tg	−62		°C
Pressure	P	4		10^7^Pa
Cavity area	τ	8		10^–11^m^2^
Peak-to-vallev distance	δ	500		nm
Peak-to-peak distance	I	587		nm
Hydraulic radius	r_*c*_	5		μ*m*
Diameter	d	10		μm

### Measure of PLC Pillars Elongation as a Function of the Demolding Temperature

The detached PCL replica from the mold was characterized by using a Dual Beam (SEM- FIB) FEI Nova 600 NanoLab system to measure the pillars deformation. Longitudinal and transverse deformations of scaffolds detached from the mold at different temperatures (25°C, 20°C, 15°C, and 10°C) were measured. For the SEM acquisition, a beam energy of 30 kV and an electron current of 98 pA were used.

### *In vitro* Cell Cultures

The fabricated scaffolds were used to carry out studies of cell growth using the THP-1 and the HS-5-GFP (transduced to express the green fluorescent protein). Control cells were seeded in standard Sigma polystyrene cell culture plate. All the scaffolds, used for the study, were first sterilized by keeping them for 2 h in 1 ml of a solution composed of 70% of ethanol (34963, Sigma-Aldrich) and at 30% of sterile DI water. Then, they were dried under a hood, washed twice in PBS and dried again. THP-1 is a human monocytic cell line derived from an acute monocytic leukemia patient and HS-5 is a human bone marrow stromal cell line. The used cells were provided from the laboratory of Medical Oncology at the University Magna Graecia of Catanzaro. The THP-1 cell line was cultured in RPMI-1640 medium with 1X L-glutamine (Gibco, Thermo Scientific) and supplemented with PMA (phorbol 12-myristate 13-acetate, 50 ng for 1 ml, Sigma Aldrich) which stimulates the differentiation of monocytes into macrophages, 10% fetal bovine serum (FBS, Gibco, Thermo Scientific, 1% penicillin, and streptomycin. The HS-5 cell line was grown in DMEM medium with 1X Glutamax (Gibco, Thermo Scientific). All cell cultures were maintained in incubator at 37°C in a 5% CO_2_ atmosphere. When the cells reached confluence, were washed in phosphate buffered saline solution (PBS), treated with trypsin-EDTA 0.25% (Gibco, Life Technologies), centrifuged (130 *g*, 5 min, 18 °C), re-suspended in fresh medium and seeded on the scaffold by using the “drop seeding” method that allow to cells to get better contact with the surface of the devices in the early stages of growth (Limongi et al., 2013).

### Evaluation of the Cell Viability on the PCL Scaffolds

Cell viability was performed by seeding the cells on fragments of four different types of scaffolds: 3D micro-structured devices with regular micropillars, 3D micro-structured scaffolds with irregular deformed pillars, 2-dimensional (2D) rough devices and 2D flat devices. Previous studies (Gentile et al., 2010) showed that cells grow preferentially on rough substrates than on smooth surfaces. On these bases, cell growth was compared between microtextured, disordered roughed and flat surfaces.

The different types of supports were all made performing the HE process and using different demolding temperatures. While, to fabricate the flat devices, a flat glass support replaced the silicon support and, after the demolding step, some of these scaffolds were sanded to get specific roughness values. The roughness of these scaffolds was measured with a profilometer (Dektak) and it is 945 nm. Each scaffold fragment was placed in a multiwell plate and for each cell line, three experimental tests were carried out. On each scaffold they were seeded about 2000 cells for THP-1 and 5000 cells for HS-5. The cultures were kept for 48 h in an incubator (37°C, 5% CO_2_). The proliferation rate was performed with the cell counting kit CCK 8 (Dojindo) in combination with 450 nm absorbance measurements on a plate reader. Data are expressed as mean ± standard error, the number of samples used in each experiment is provided in figure legend and statistical comparison between two measurements were made using *t*-tests.

### Evaluation of the Cell Adhesion on the PCL Scaffolds

#### HS-5-GFP Fluorescence Microscopy

To verify cell adhesion on the four devices, each scaffold was loaded with 50000 cells and, after 48 h of incubation, placed on a glass slide and washed twice in PBS. Following the washes, cells were fixed with 4% paraformaldehyde for 12 min. They were then washed two times in PBS, then permeabilized by applying 0.1% Triton in PBS for 15 min and again washed two times in PBS. Finally, the staining was performed with DAPI for 30 min. The green GFP fluorescence was just present since this cell line was previously engineering. Cell were observed by a fluorescence microscope (Nikon Eclipse Ti) and the images were then processed using the ImageJ software.

#### THS-5-GFP and THP-1 Scanning Electron Microscopy

SEM images of both cell lines, cultured for 48 h, were acquired. To verify cell adhesion on the four devices, each scaffold was loaded with 50000 cells and, after 48 h of incubation, placed on a glass slide and washed twice in PBS. The samples were washed with PBS and then fixed using a solution of 1.2% glutaraldehyde (G5882 Sigma Aldrich) in 0.1 M sodium cacodylate (C0250 Sigma Aldrich), for 1 h at room temperature. After the fixing, the cell supports were washed with sodium cacodylate buffer solution (0.1 M, pH 7.4), subsequently these were treated for 1 h with a solution of 1% osmium tetroxide (CAS #20816-12-0, 19110, Electron Microscopy Sciences) in 0.1 M sodium cacodylate. Subsequently, three washes in distilled water were performed, followed by washes of 5 min each in increasing concentrations of ethanol (30%, 50%, 70%, 80%, 90%, 96% v/v). After that, the cell supports were washed two times for 15 min each in a 100% ethanol solution, followed by gradual replacement of ethanol with the hexamethyl-disilazane (HMDS, 379212, Sigma Aldrich). To perform this step, the samples were first immersed in a solution of ethanol/HMDS at a ratio of 3:1, for 10 min; then at a ratio of 1:1 for 10 min; and at a ratio of 1:3 for 30 min. Finally, the samples were left under a hood to let the solution evaporate completely and sputtered with gold for the SEM visualization. The scaffolds were observed in a Dual Beam (SEM- FIB) FEI Nova 600 NanoLab system. For the SEM acquisition, a beam energy of 30 kV and an electron current of 98 pA were used.

## Results and Discussion

### Demolding Force Analysis: Comparison Between the Analytical Molds and the Experimental Results

The comparison between the experimentally measured demolding force and that resulting from the calculation with the analytical model are shown in the left panel of Figure 3 as a function of the demolding temperature. The temperature range was chosen, for practical reasons, between 0°–25°C, which is easy to be achieved and maintained. In addition, above and below the chosen range, the experimental results did not give new information since they did not consistently change (data not shown). The data show a good fit between the model and the experiments (*p* < 0.05), with a slight margin of error, probably due to the approximations made in the model. If the demolding temperature decreases, the demolding force decreases as well in the range of temperature considered, for the specific used material, and microstructures replicated. The model allowed calculating the proper value of demolding temperature by which we obtained a value of demolding force correspondent to tensions lower than the material strength. For such value of temperature, it was possible to obtain regular and not deformed PCL microstructures.

**FIGURE 4:**
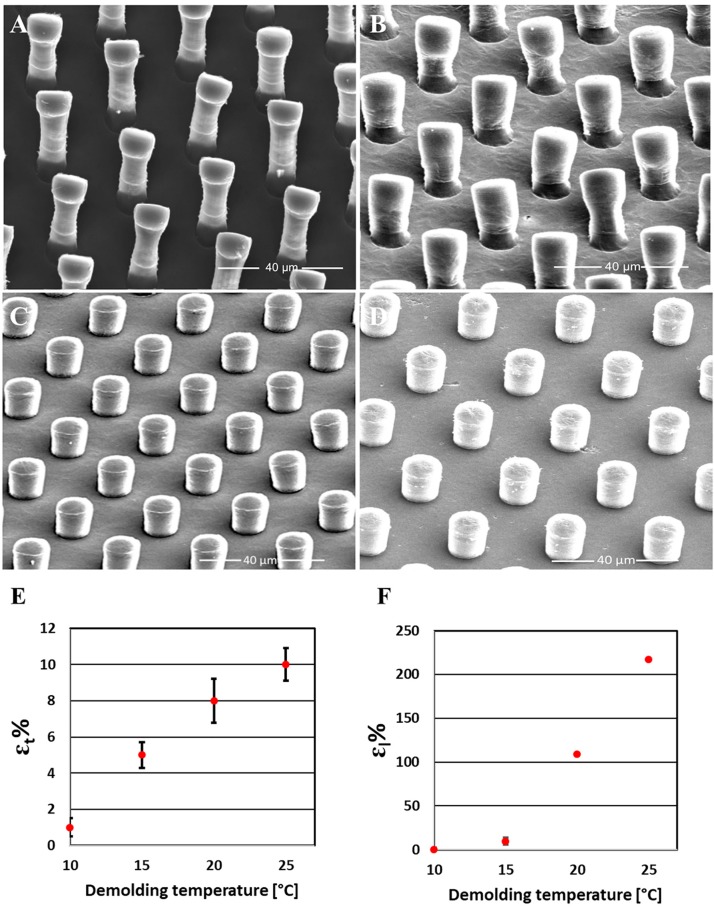
Measurements of the pillars elongation as a function of the demolding temperature. SEM images of PCL scaffolds detached from the mold at 25°C **(A)**, 20°C **(B)**, 15°C **(C)**, and at 10°C **(D)**; Quantification of transverse **(E)** and longitudinal deformation **(F)** of the pillars {The deformed height (Hd) and width (Wd) of the pillars fabricated by using different demolding temperatures were measured from SEM images and compared with the nominal height and width of the pillars. The longitudinal deformation in percentage (εl%) was calculated by [(Hd-Hi)/Hi] × 100 and the transverse deformation in percentage (εt%) was calculated by [(Wd-Wi)/Wi] × 100}.

The scaffolds obtained by the HE process were observed by means of an optical (Figures 3A–D) and a scanning electron microscope (Figures 4A–D). Both the transverse and longitudinal deflections of the pillars were measured. The graphs (Figures 4E,F) show that there is a growth of pillars deformation with increasing demolding temperature. In particular, the SEM images show that the detached scaffolds at 25°C (Figure 4A) have damaged pillars, in fact, they appear slightly bended and stretched. While, those separated from the mold at 10°C (Figure 4D) exhibit microstructures in good conditions, reproducing the same shape of the mold micro cavities. The scaffolds detached at 20°C (Figure 4B) and 15°C (Figure 4C) showed slightly lower deformation of those obtained at 25°C. Therefore, it is possible to conclude that the best result can be obtained by detaching the scaffolds from the mold at 10°C. The results can be interpreted considering that, lowering the demolding temperature, the demolding force and so the stretching tension at the interface between the mold and the replicas, decreases as well, at values lower that the material strength. In this condition, the micropillars will not be deformed. However, we observed that the detached samples at the lower temperatures have pillars in good condition in the center area of the scaffolds, while in the side areas some pillars show anyway signs of stretching. This is probably due to the uneven distribution of the applied tensile force through the tweezer. In addition, the laser thermometer was focused on the PCL scaffold and measured the temperature on the material surface. Due to heat flux, the temperature in the internal part of the scaffold might be different and this might explain the deviation between the experimental points and the theoretical model, and the different deformation obtained between the central and the external part of the replica. This aspect is crucial especially when large areas are hot-embossed.

### Metabolic Rate of the Cells Seeded on the PCL Scaffolds

**FIGURE 5:**
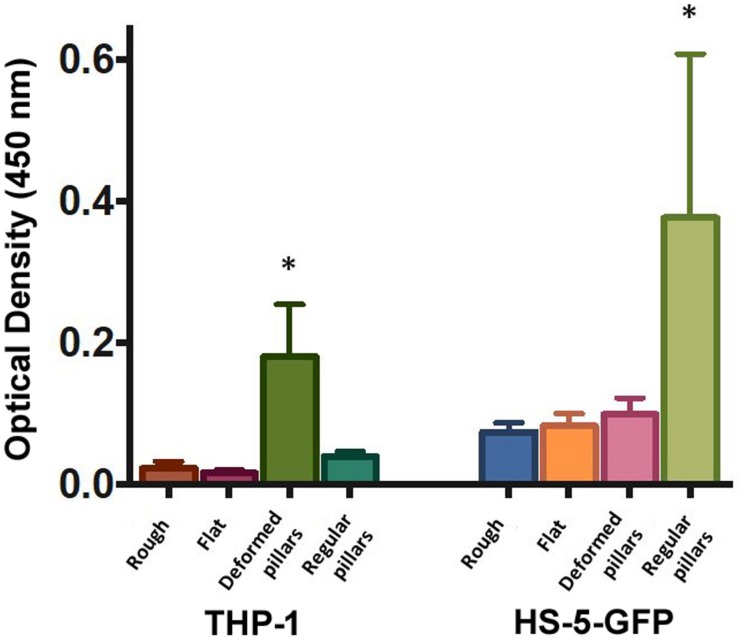
Cell viability as measured by the WST-8 assay using: THP-1 and GFP-HS-5 cells after 48 h of incubation on rough (Rough), flat (Flat), PCL surfaces and integrating regular (Regular pillars) and deformed micropillars (Deformed pillars). Data were expressed as mean ± standard error (*n* = 3). ^∗^Represents the statistically significant difference when the group is compared with the other of the same cell line (*p* < 0.05).

The viability data of cells seeded on PCL scaffolds (rough, flat, deformed, and regular pillars) after 48 h of incubation are plotted in Figure 5. Control data of cells growth on standard polystyrene cell culture plates are not shown since they confirmed that control cells were healthy and vital for both lines. Cells viability has been analyzed considering how much each line preferred one growth substrate to the other and not comparing the growths on the two lines. In the figure, it can be observed both cell types have grown preferentially in the presence of micro-structured scaffolds, rather than on flat and rough unstructured ones. It is interesting to observe, that THP-1 cells viability is significantly higher when cells ware seeded on deformed pillars, while considering HS-5 cells, it is significantly higher where seeded on PCL surface with regular pillars.

### Fluorescent and Scanning Electron Microscopy Analysis of Cells Growth on the 3D PCL Scaffolds

**FIGURE 6:**
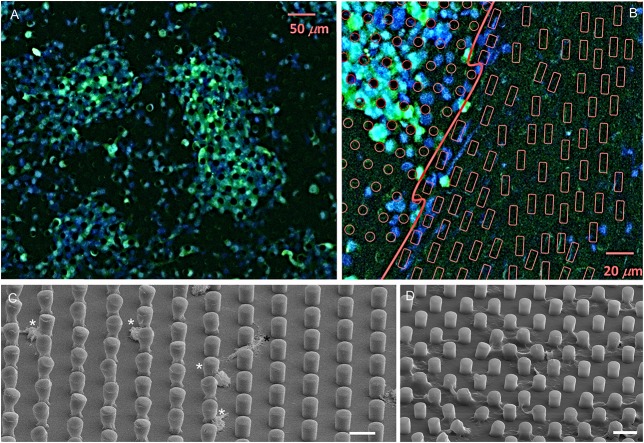
Fluorescence microscope images, low magnification **(A)** and high magnification **(B)**, of GFP-HS-5 cells cultured on a PCL scaffold. In B, regular pillars are evidenced with red circles while deformed ones with red rectangle. SEM images of GFP-HS-5 **(C)** and THP-1 **(D)** cells grown on PCL scaffolds presenting both areas with regular and deformed micropillars; scale bars 20 μm. White asterisks: dead cells; black asterisk: healthy cell.

Fluorescent and scanning electron microscopy (Figure 6) allowed a quick and efficient qualitative analysis of cells survival and distribution after seeding on the PCL scaffold with both regular and deformed micropillars. Figure 6A shows low magnification fluorescence microscope images of HS-5-GFP cells seeded on a 820 × 780 μm^2^ scaffold. Here, considering GFP fluorescence (green) and nuclear DAPI signal (blue) it is possible to note that cells prefer grow on the uniform and regular 3D structures (higher cellular density region in Figure 6A and red circles in high magnification panel 6 B).

SEM analysis confirm that HS-5 are they are vital and very well attached to the substrate of the more regular areas (black asterisk) while they are less viable or dead (white asterisks) in areas with irregular pillars (Figure 6C). Unlike THP-1 cell, prefer to grow on the areas of the most irregular or deformed substrates (Figure 6D).

It is known, as reported in scientific literature (Gentile et al., 2010) that the mechanisms of differentiation and cell reproduction appear to be influenced by the presence of a solid support realized using different kinds of biomaterial and design. In fact, while cells look for the suitable substrate for their anchorage and grow adherent to it, nano- and micro-topography of their surface stimulate changes in the behavior of cells and play an important role in the regulation of their proliferation, vitality and differentiation. Based on these observations, we can justify the experimental results by considering the physiological function of the used cell lines. Indeed, macrophages (THP-1) are cells involved in the cicatricial/inflammatory processes, and are recruited from peripheral blood in places where the tissue presents damages (Tsuchiya et al., 1980) consistent to the preference of THP-1 for irregular surfaces (McWhorterChase et al., 2015; Li et al., 2018). These cells are healthy at a density range of 1 × 10^5^–2 × 10^6^ cells/ml, if they get denser they may slow down or stop dividing and may clump or have an irregular or blubbing appearance (Stokes and Doxsee, 1999). Stromal cells line as immortalized human bone marrow stromal cell line, HS5, can grow easier on regular microstructures replicated with a constant pitch, according to their function to form connective tissue (Roecklein and Torok-Storb, 1995; Pitari et al., 2015).

## Conclusion

The possibility of building complex tissues, *in vitro*, is a frontier of great importance. For the achievement of this objective, we demonstrated effective implementation of appropriate scaffolds, integrating a regular array of micro pillars on their surface. In particular, we used HE on silicon microstructured molds for the fabrication of microfeatured PCL scaffolds. In addition, an analytical model was developed, which allowed calculating the demolding force in a certain range of temperatures and can be applied as an aiding tool to estimate the proper process parameters for obtaining defect-free microstructures. The analytical model was validated by an experimental set-up, which allowed to measure the demolding force, during the detachment of PCL scaffolds from the silicon mold. We demonstrated that a crucial parameter that influences the fabrication result is the temperature during the demolding process. Using temperatures of ten centigrade during the demolding of PCL scaffolds, we obtained micro-structures in terms of size and shape, reproducing the mold microstructures. Finally, we performed experiments of cell proliferation on these scaffolds to demonstrate how crucial the control of the dimensions and regularity of microtextured scaffolds is. In fact, performing the cultivation of cells on the substrates, it was possible to observe how, according to the specific type of cell, they prefer to adhere to regular patterns or to unregularly microstructured surfaces. In particular, the stromal cell lines (HS-5) showed to grow on regular microstrucured surfaces, while the lymphocyte cell lines (THP-1) showed a preference toward irregular microstructured surfaces.

## Data Availability Statement

All datasets generated for this study are included in the article/Supplementary Material.

## Author Contributions

TL and GP: conceptualization. EL and CB: methodology. BS, MG, MCu, and CC: validation. PC and MCo: formal analysis. ED and BS: investigation. MCo: data curation. GP: writing and original draft preparation. ED, CC, SP, GP, and TL: writing, review, and editing. GP, AF, ED, EL, PTs, and PTg: supervision. All authors provided approval for the publication of the content.

## Conflict of Interest

The authors declare that the research was conducted in the absence of any commercial or financial relationships that could be construed as a potential conflict of interest.
