# Supplementary material for: Influence of the Fabrication Accuracy of Hot-Embossed PCL Scaffolds on Cell Growths
Source: Front Bioeng Biotechnol. 2020 Feb 14;8:84. doi: 10.3389/fbioe.2020.00084 (PMC7033415; doi:10.3389/fbioe.2020.00084)
Supplement: Supplementary file 1 [file Table_1.DOCX]

Supplementary Material

# Heat conduction between the mold and the PCL scaffold

The simulation program Comsol Multiphysics ver. 5.1 (Heat transfer module) has been used to evaluate thermal conduction in the aluminum and silicon molds and the PCL replica. The model was a 2D representation of the experimental setup consisting of three different domains: an aluminum layer (60 x 35 mm), a silicon layer (20 x 0.5 mm) and a PCL layer (10 x 4 mm). All the domains have been considered as homogeneous structures.

We performed two different simulations. In the first simulation, the right and left edges of the aluminum domain were set to the fixed temperature of 23°C while the upper and lower edges were set to 68°C. Initial temperature values for all domains were set to 23°C. In the second simulation, all the edges of the aluminum domain were set to the fixed temperature of 0 °C. Initial temperature values for all domains were set to 68°C. In both cases, we investigated the heat conduction as a function of time. The following Figure shows the sketch used for the simulations.


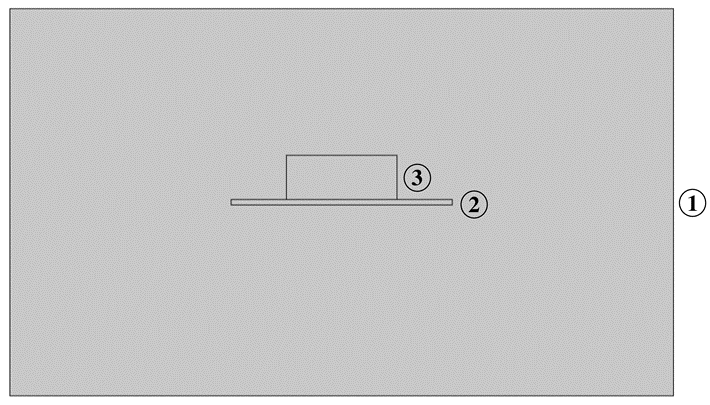


**Figure 1.** Sketch of: 1) aluminum mold; 2) Silicon mold; 3) PCL replica.

Material data are presented in the following Table.

Table 1. Properties of the materials used during the simulation.

| Material | ρ [Kg/m^3^] | k [W/(m∙K)] | Cp [J/(Kg∙C)] | Reference |
| --- | --- | --- | --- | --- |
| Aluminum | 2700 | 238 | 900 | Comsol library |
| Silicon | 2329 | 130 | 700 | Comsol library |
| PCL | 1145 | 0.1 | 1400 | [REF] |

[REF] BB Naghshine, JA Cosman, and A Kiania. Synthesis of polycaprolactone-titanium oxide multilayer films by nanosecond laser pulses and electrospinning technique for better implant fabrication. Journal of Applied Physics 120, 084304 (2016)

The mesh was extremely fine for the entire geometry (15610 triangular elements).

In the following pictures, the values of the temperature as a function of the time of a specific point of the aluminum mold and in an area close to the microstructures are reported.


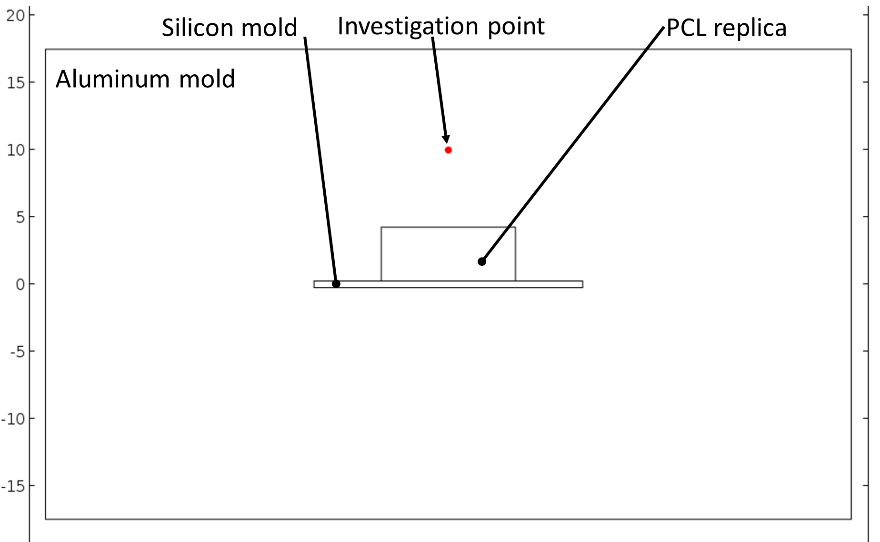


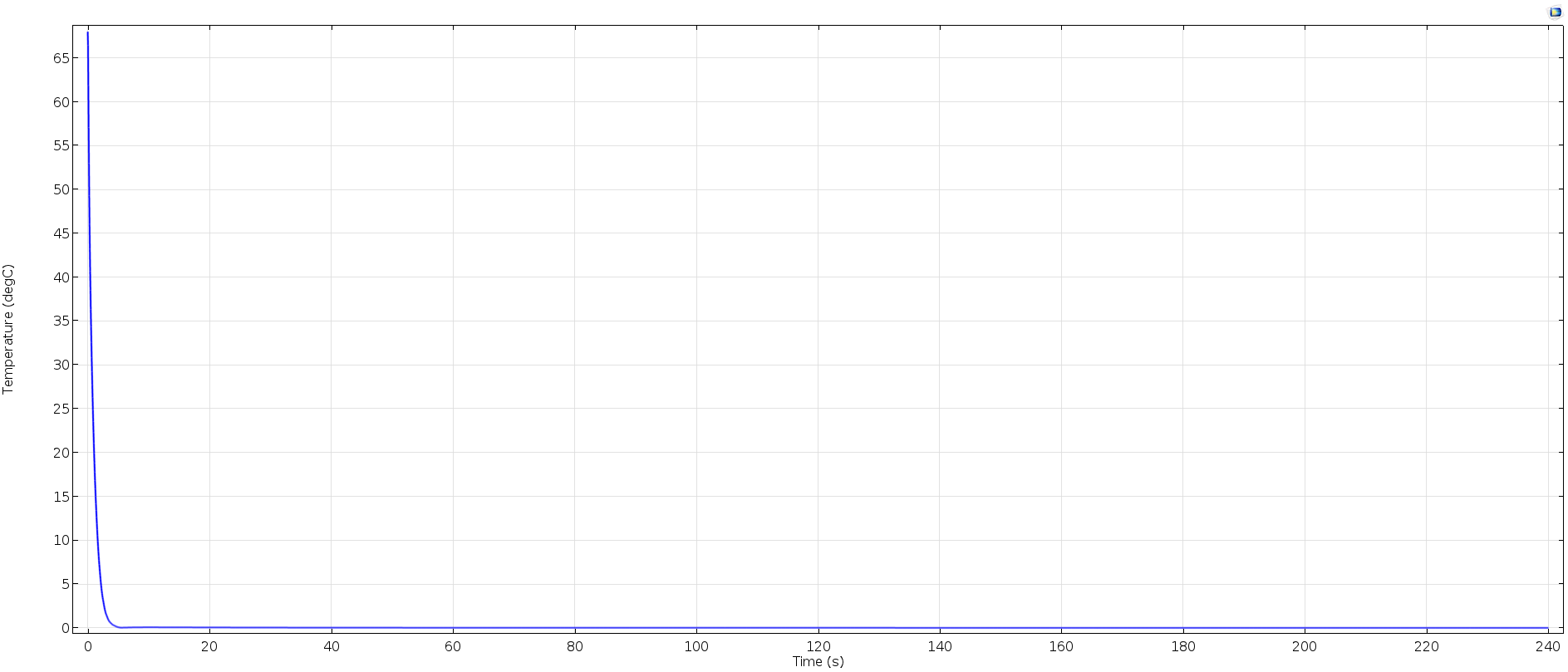


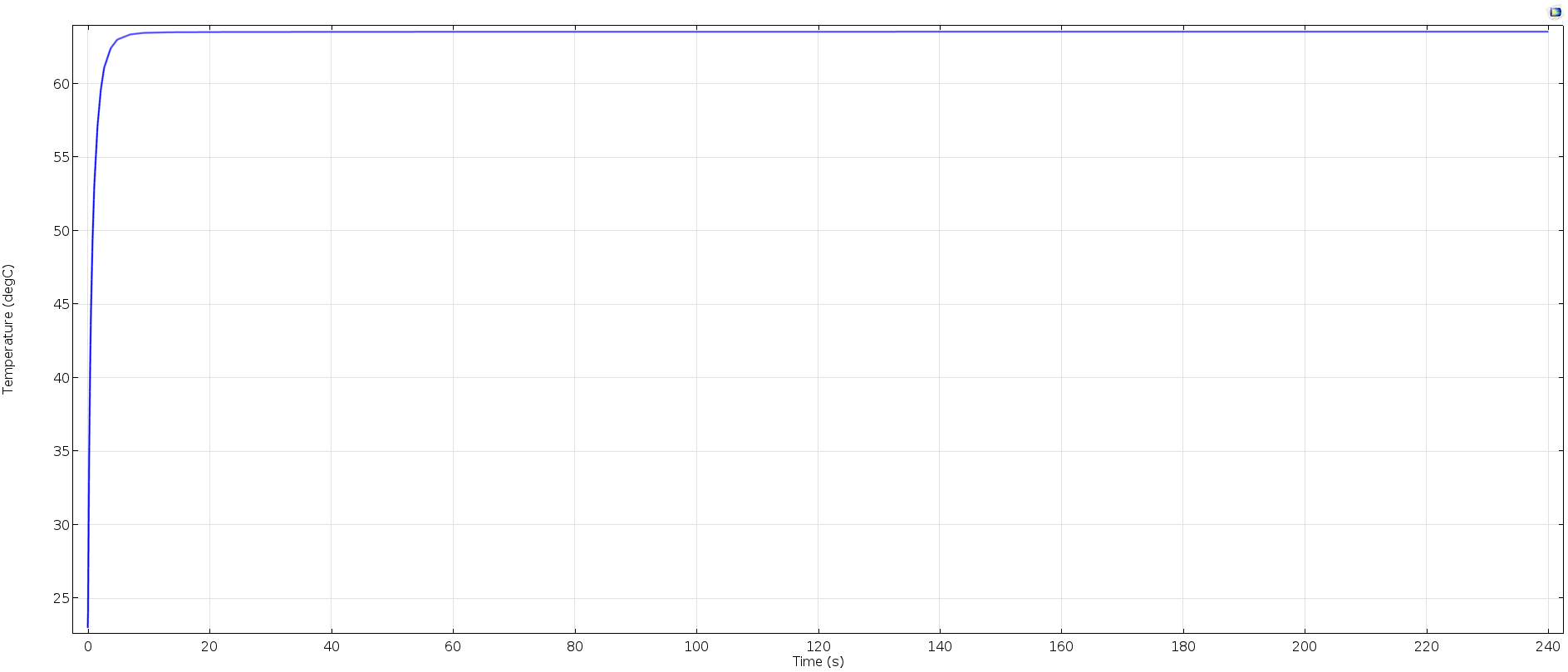


**Figure 2.** Top: Sketch of the point in the aluminum mold in which the temperature is investigated as a function of time; Middle: temperature as a function of time during the cooling process; Bottom: temperature as a function of time during the heating process.


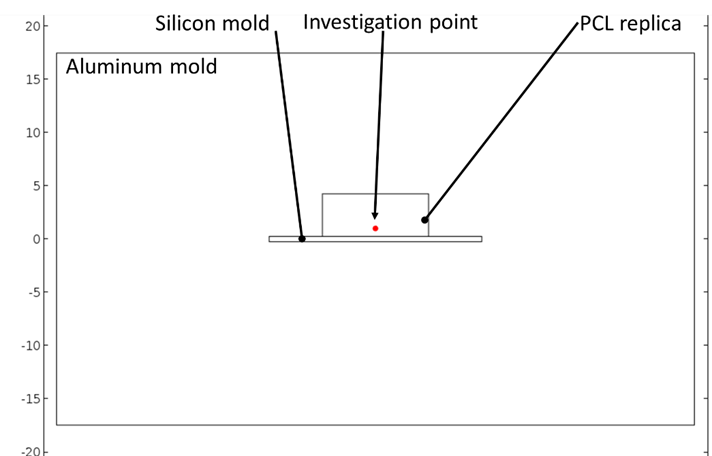


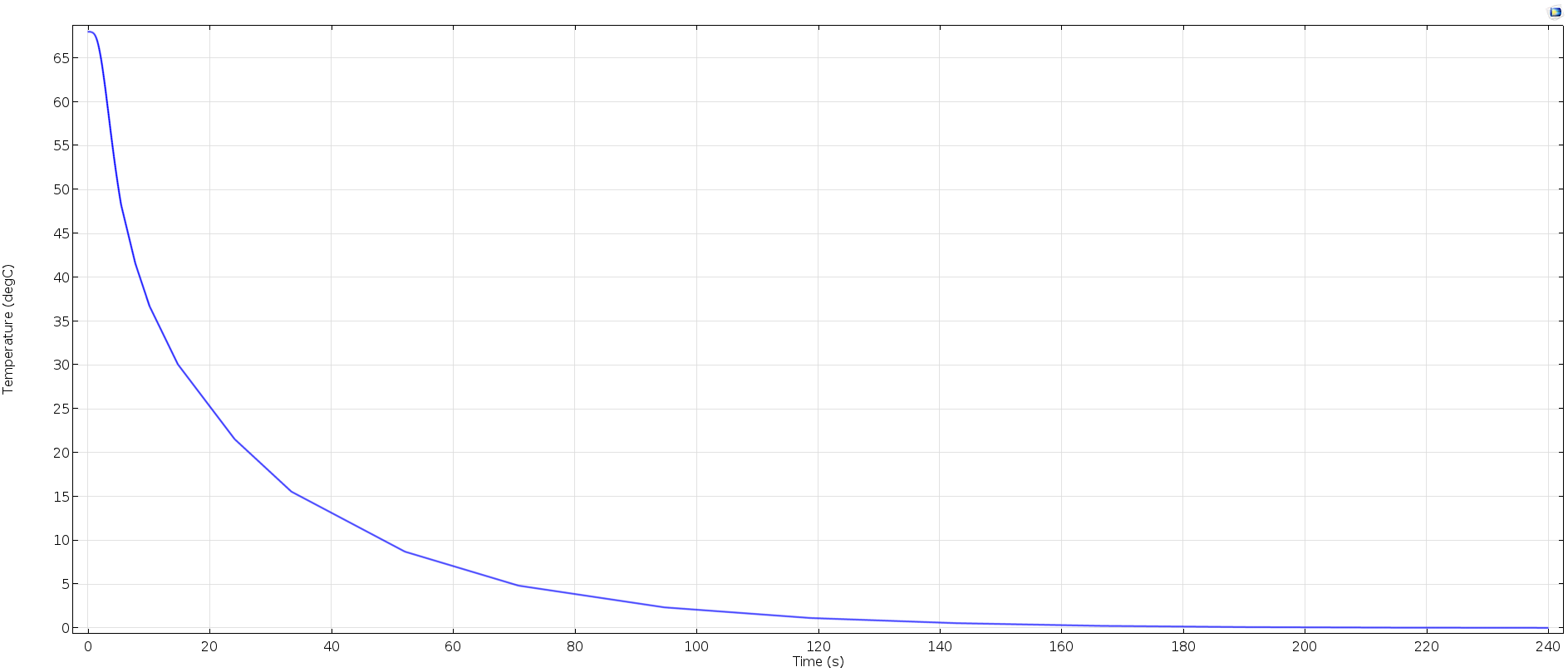


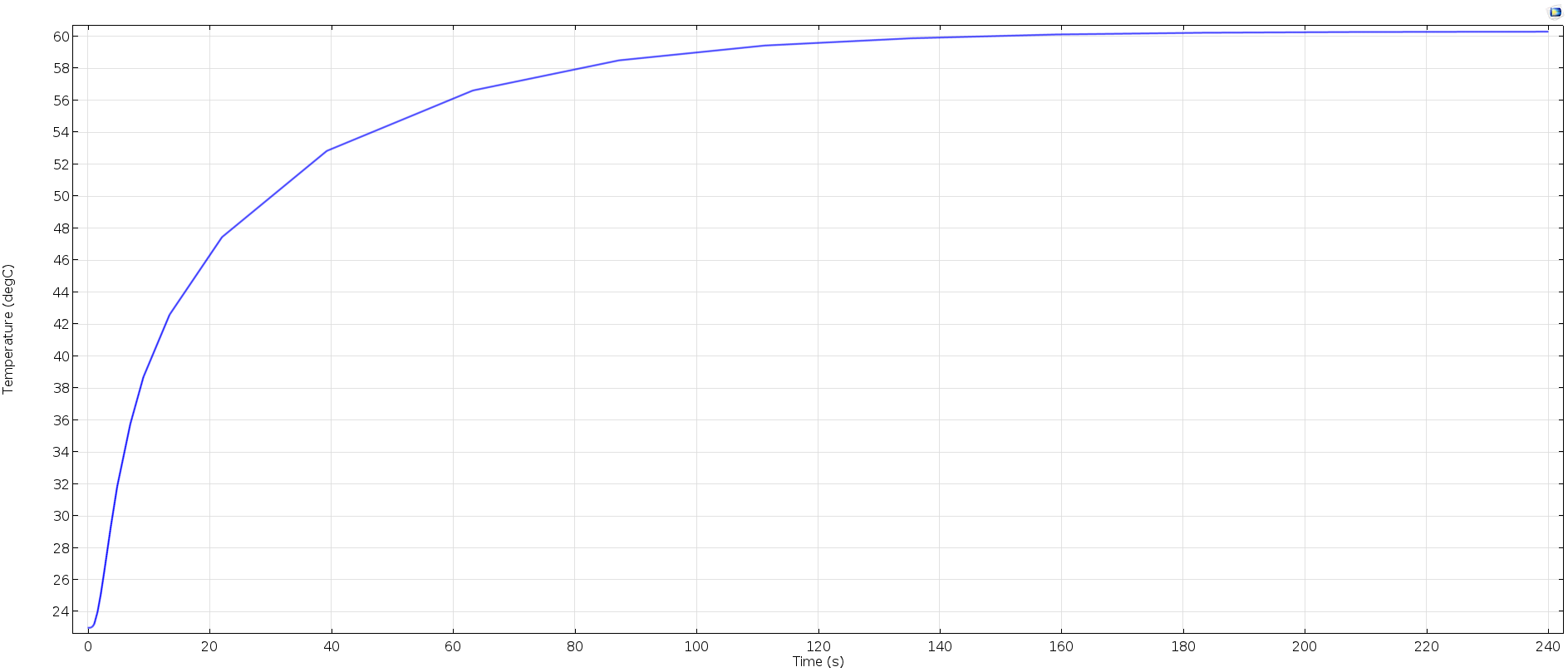


**Figure 3.** Top: Sketch of the point in the PCL replica in which the temperature is investigated as a function of time; Middle: temperature as a function of time during the cooling process; Bottom: temperature as a function of time during the heating process.


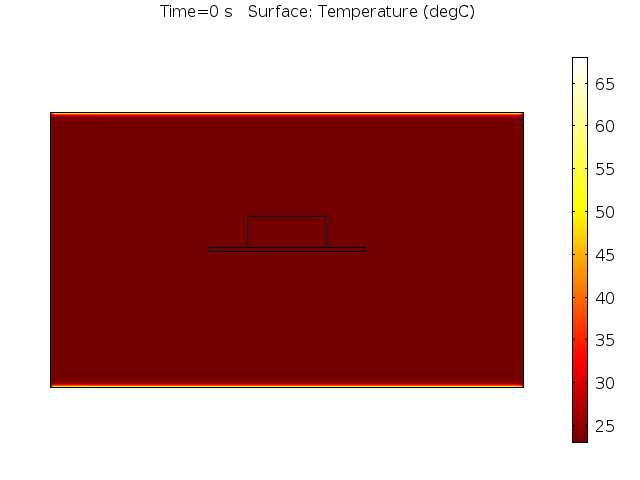

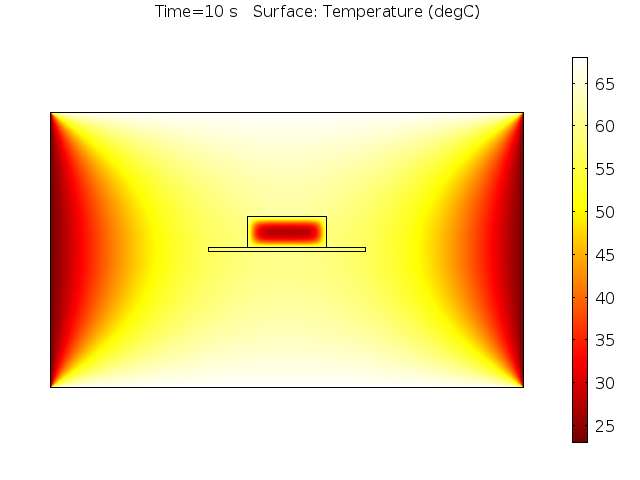

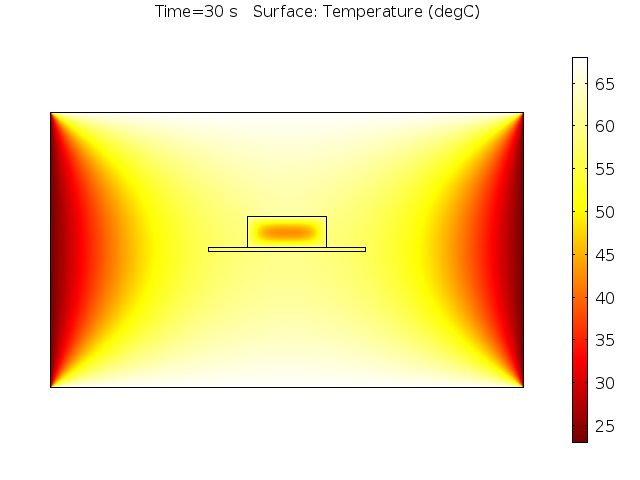

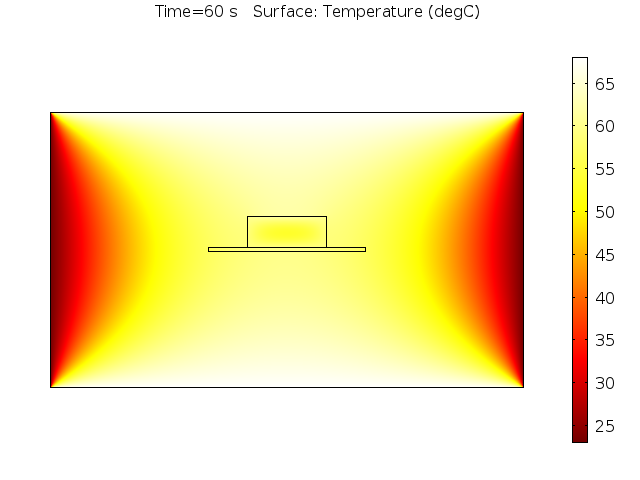

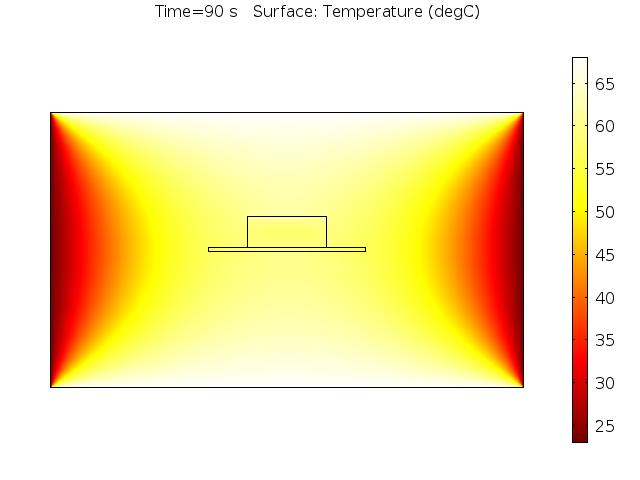

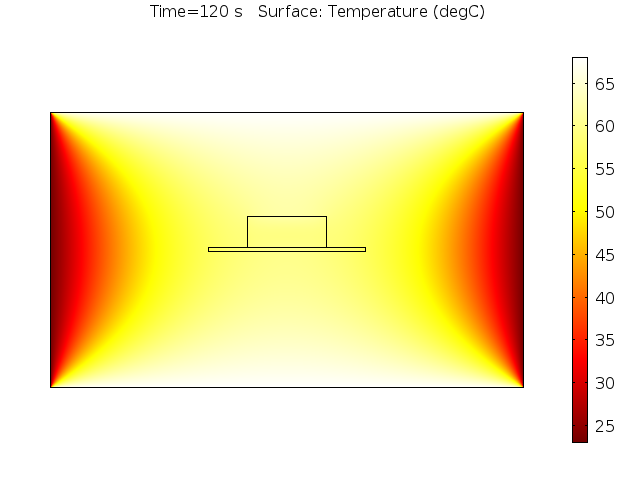

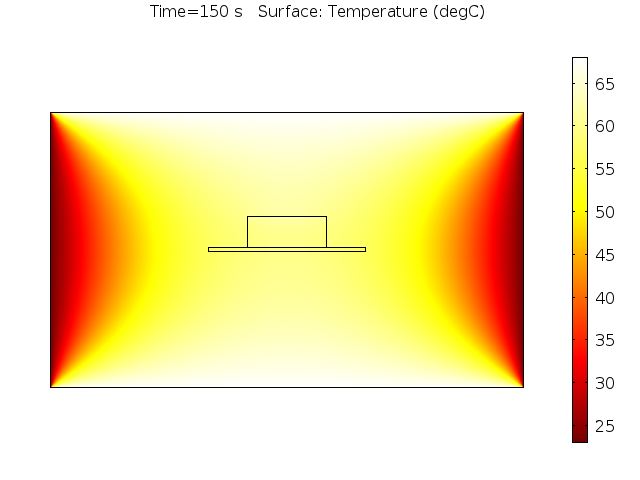

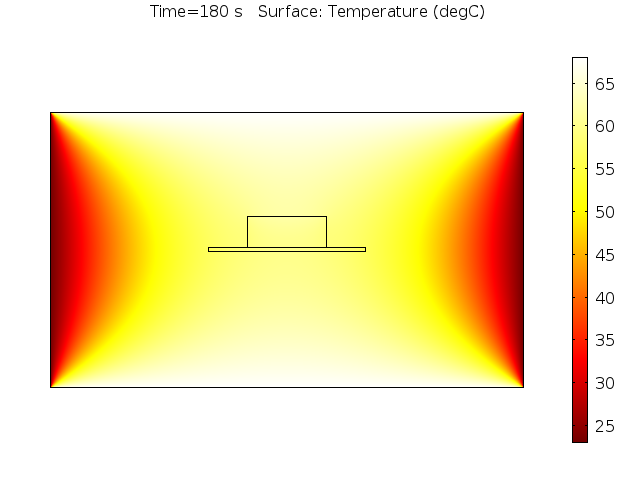

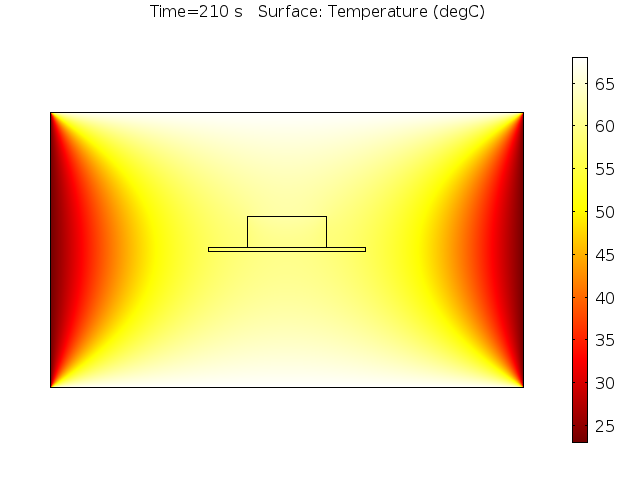

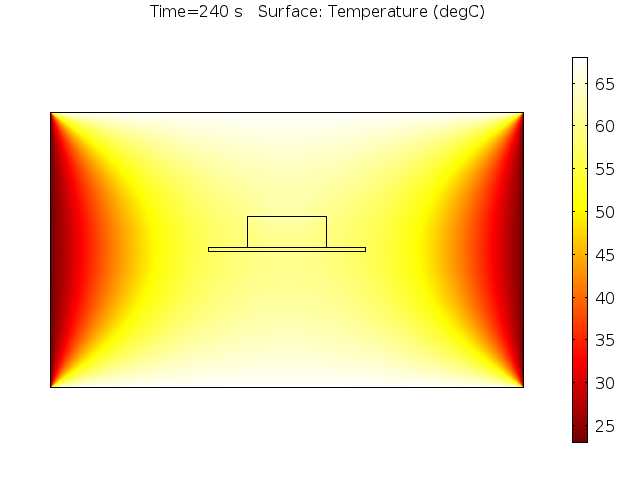


**Figure 4**. Temperature as a function of the time in the full model during the heating process.


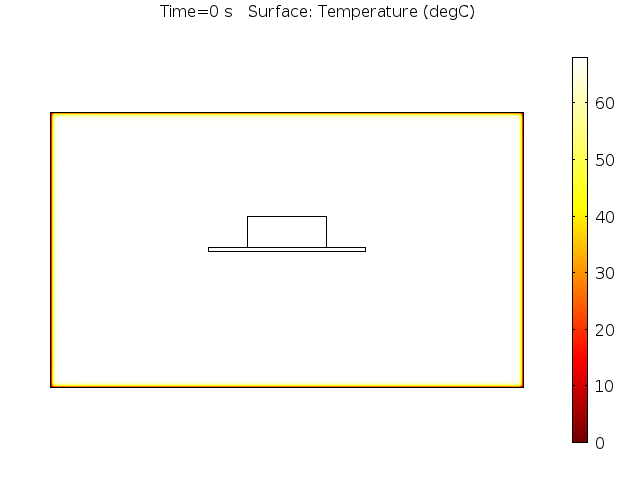

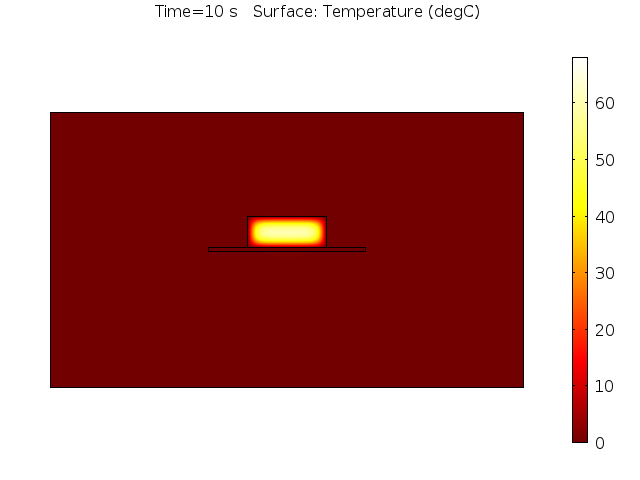

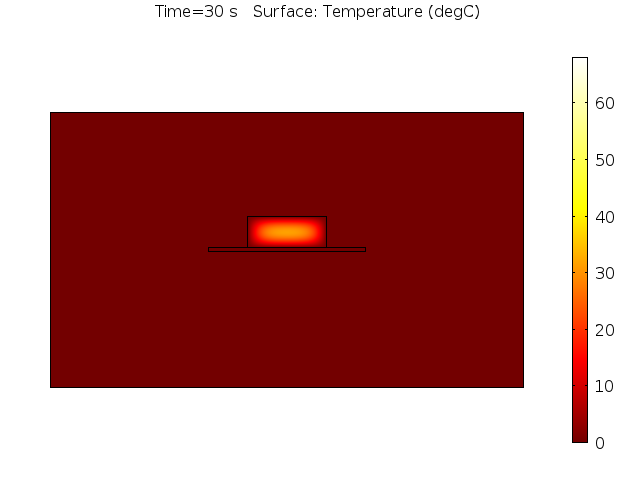

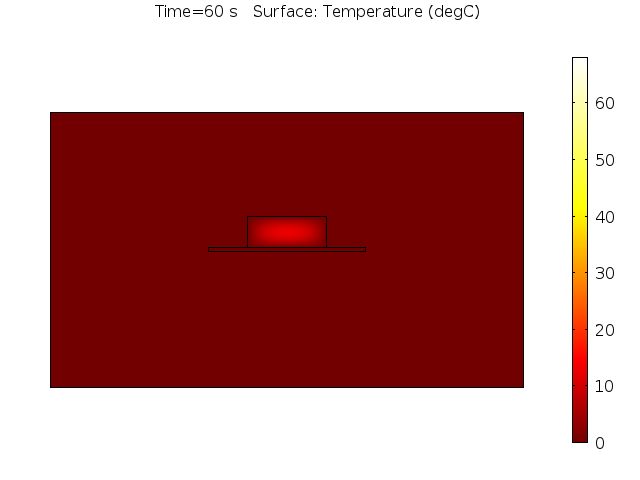

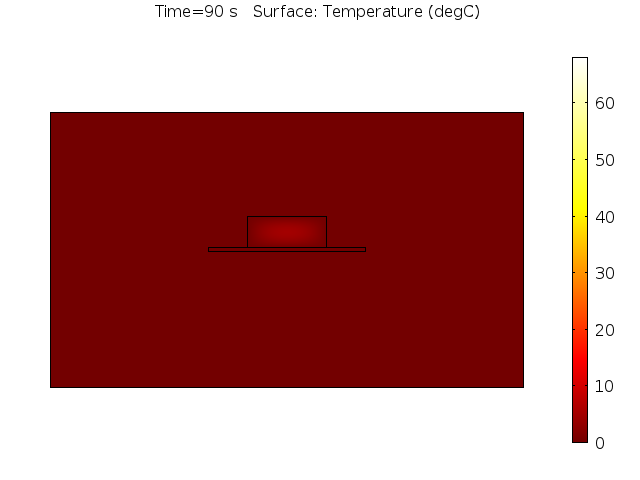

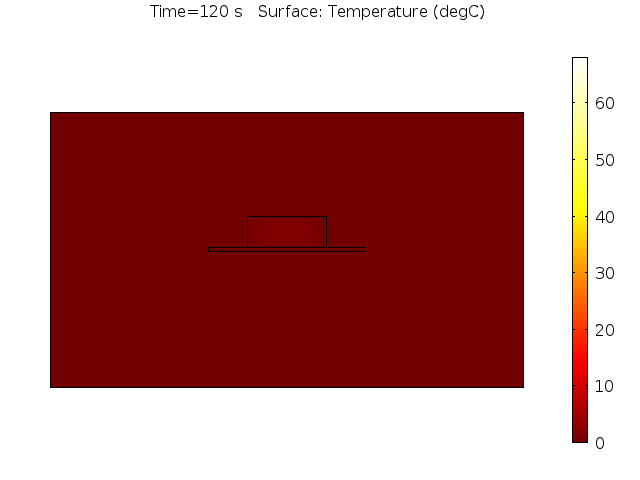

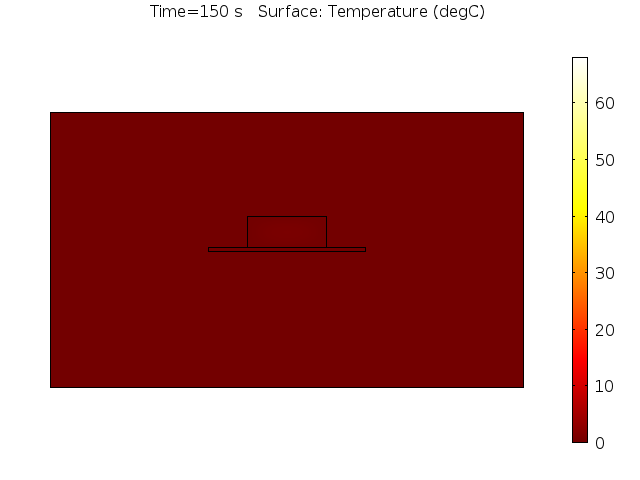

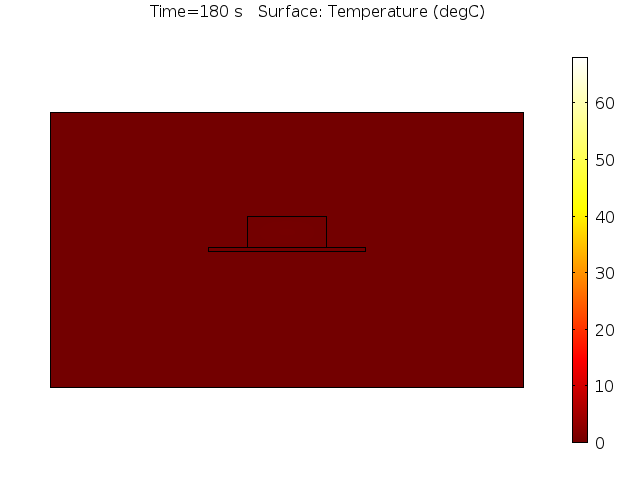

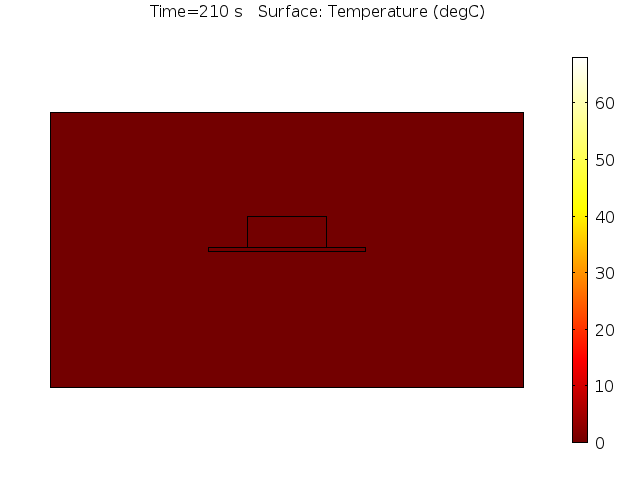

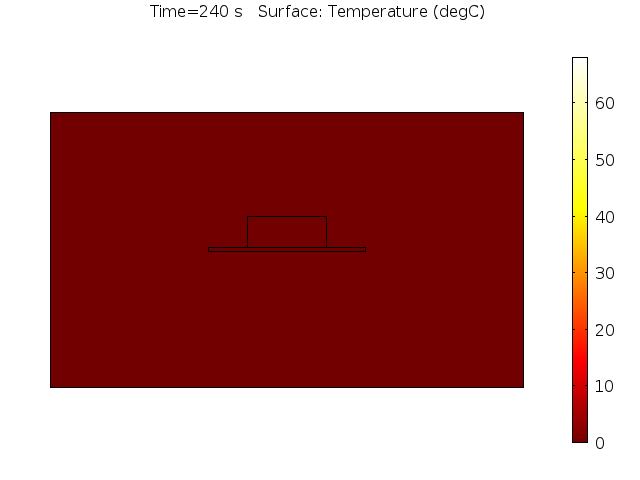


**Figure 5**. Temperature as a function of the time in the full model during the heating process.

# Distribution of the pillar height along the PCL replica

The distribution of the pillar height along the PCL replica was investigated by SEM analysis. In particular, as shown in Figure 6, pillars height have been evaluated with respect to the center (position 0) and by moving on the sample with a step-size of 1 mm on the left (negative positions) and on the right (positive positions). For each position, an area of 100x100 µm2 (magnification of 2000X) was analyzed for the evaluation of pillars height (tilt angle of 45°). SEM acquisitions were obtained at 10 kV with a probe current of 50 pA.


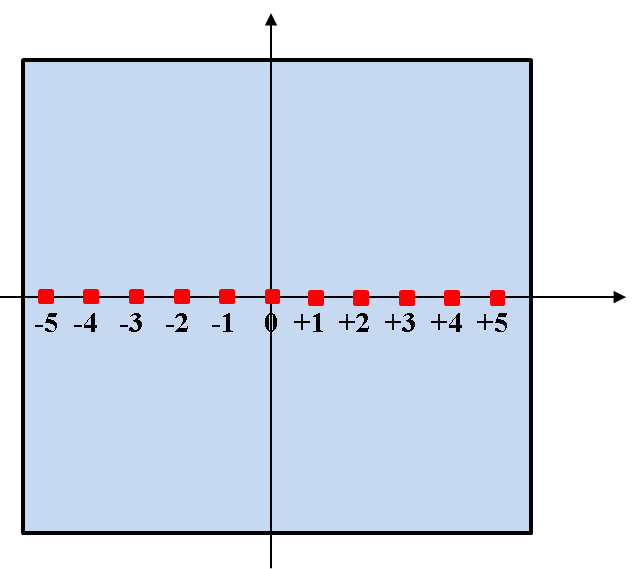


**Figure 6.** Acquisition pattern for the characterization of pillars geometry.

In Figure 7, the distribution of average pillars height is reported. The effect of the fabrication process resulted in a parabolic-like pillars height profile by moving from the center to the external part of the sample. The parabolic profile is characterized by lower pillars height at the center, while they become higher on the external part of the sample. The deformation is relevant in the last 2-3 mm of the replica. This issue could be solved by fabricating a bigger replica with a sacrificial area 2-3 mm wide without pillars.


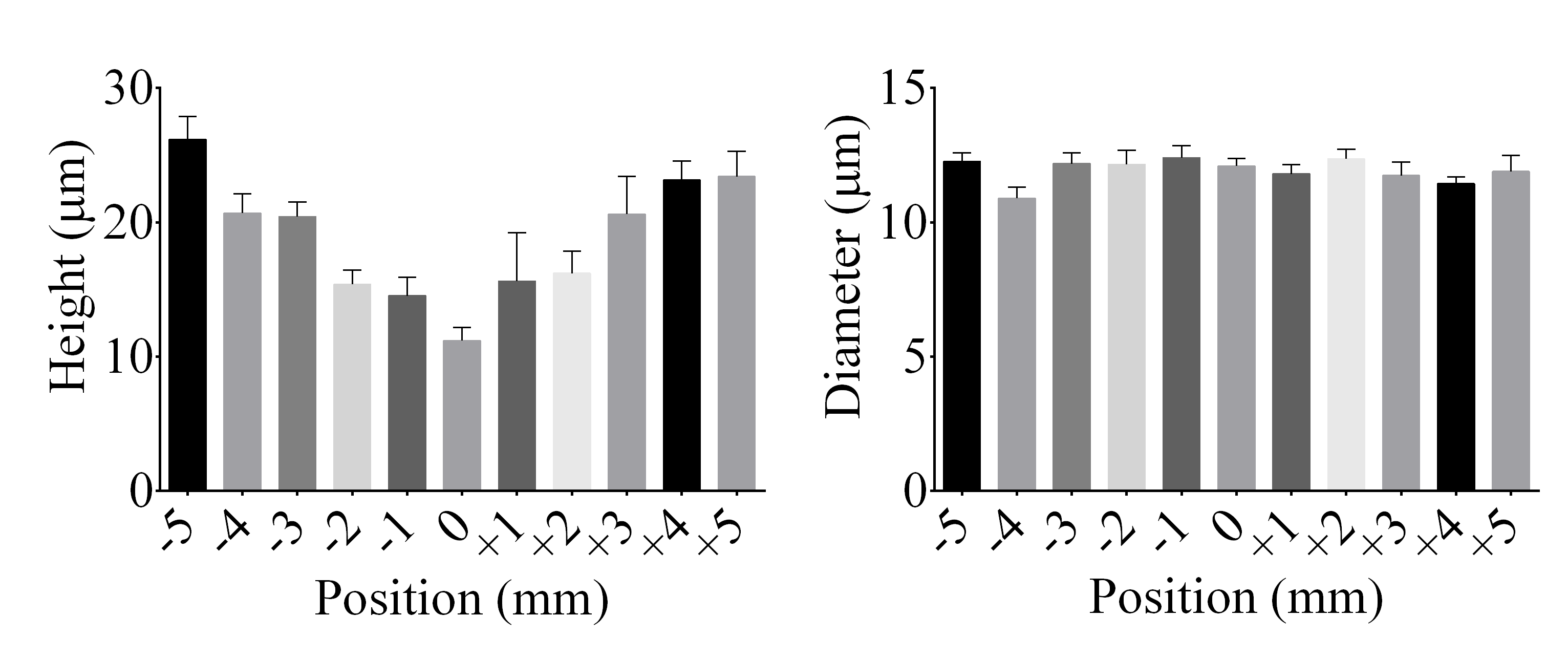


**Figure 7.** Average pillars height (left) and diameter (right).

# Measurement of the demolding force

The experimental set-up consisted of a tweezer reproducing a lever of class 1. The tweezer integrated a holder where the replica could be fixed on one arm of the tweezer and the mold on the other arm on the same side. By applying a pressing force on the arms of the opposite side of the tweezer, the mold and the replica could be detached from each other. The fulcrum of the lever was designed in such a way to have a ratio between detaching and pressing force of 1:10. So that we could recreate a system, which was ten times more sensible than the force sensor connected to the tweezer (Figure 8A).


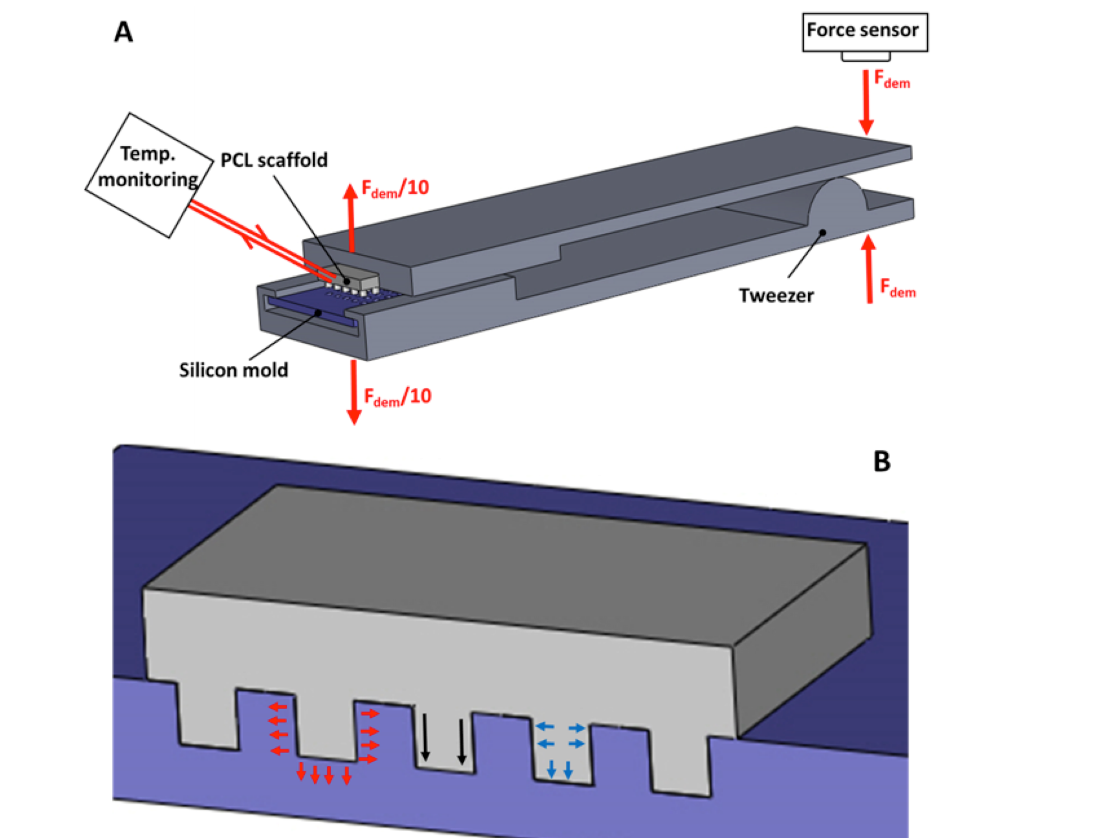


**Figure 8.** Scheme of the experimental set-up used for measuring of the demolding force (A); Scheme of the tensions acting between the replica and the mold due to adhesion (red arrows), friction (black arrows), thermal expansion (blue arrows) (B).
